# Tertiary Alkylamines as Nucleophiles in Substitution Reactions at Heteroaromatic Halide During the Synthesis of the Highly Potent Pirinixic Acid Derivative 2-(4-Chloro-6-(2,3-dimethylphenylamino)pyrimidin-2-ylthio)octanoic Acid (YS-121)

**DOI:** 10.3390/molecules161210013

**Published:** 2011-12-05

**Authors:** Matthias Gabler, Manfred Schubert-Zsilavecz

**Affiliations:** Goethe-University Frankfurt, Institute of Pharmaceutical Chemistry, Max-von-Laue-Str. 9, D-60438 Frankfurt/M., Germany

**Keywords:** YS-121, pirinixic acid, reaction improvement, nucleophilic aromatic substitution reaction at heteroaromatic halide by tertiary alkylamines, byproduct formation mechanism

## Abstract

YS-121 [2-(4-chloro-6-(2,3-dimethylphenylamino)pyrimidin-2-ylthio)octanoic acid] is the result of target-oriented structural derivatization of pirinixic acid. It is a potent dual PPAR*α*/*γ*-agonist, as well as a potent dual 5-LO/mPGES-1-inhibitor. Additionally, recent studies showed an anti-inflammatory efficacy *in vivo*. Because of its interference with many targets, YS-121 is a promising drug candidate for the treatment of inflammatory diseases. Ongoing preclinical studies will thus necessitate huge amounts of YS-121. To cope with those requirements, we have optimized the synthesis of YS-121. Surprisingly, we isolated and characterized byproducts during the resulting from nucleophilic aromatic substitution reactions by different tertiary alkylamines at a heteroaromatic halide. These amines should actually serve as assisting bases, because of their low nucleophilicity. This astonishing fact was not described in former publications concerning that type of reaction and, therefore, might be useful for further reaction improvement in general. Furthermore, we could develop a proposal for the mechanism of that byproduct formation.

## 1. Introduction

**Figure 1:**
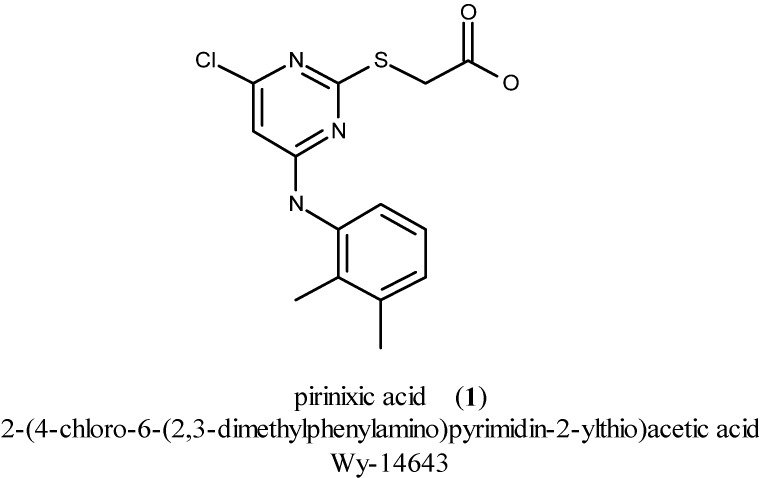
Chemical structure of pirinixic acid.

Pirinixic acid [**1**, Wy-14643, 2-(4-chloro-6-(2,3-dimethylphenylamino)pyrimidin-2-ylthio)acetic acid; Figure 1] was discovered in the 1970s, during the development of new agents against hypercholesterolemia [1]. Later on, in 1977, it was identified to be a peroxisome proliferator [2]. After the discovery of the peroxisome proliferator-activated receptor (PPAR) in 1990 [3], the agonistic activity of pirinixic acid at PPAR*α* and PPAR*γ* was detected within the next ten years [4,5].

**Figure 2:**
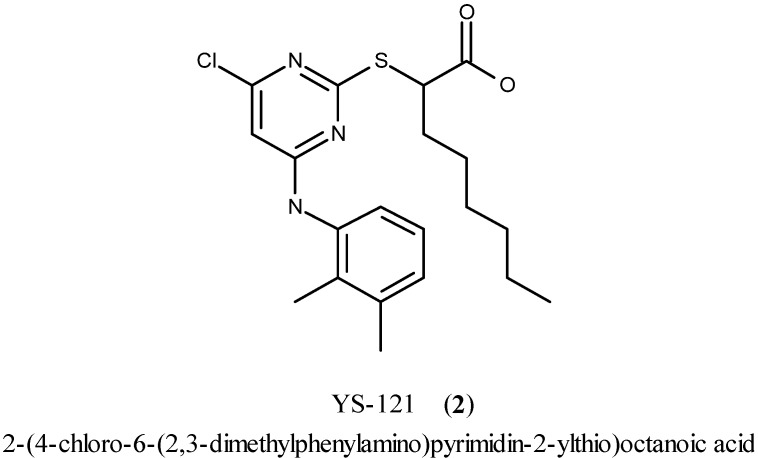
Chemical structure of YS-121.

YS-121 [**2**, 2-(4-chloro-6-(2,3-dimethylphenylamino)pyrimidin-2-ylthio)octanoic acid; Figure 2] is the result of target-oriented structural derivatization of pirinixic acid [6]. In YS-121 a hexyl residue was introduced in the position *α* to the carboxylic acid group of pirinixic acid. That structural modification enhanced PPAR*α* and PPAR*γ* agonism to values in the low micromolar range [EC_50_ = 1.0 µM (*α*) and 3.6 µM (*γ*) in a cell-based luciferase reporter gene assay] [6].

In addition to its dual agonistic activity at PPAR*α*/*γ*, further research efforts revealed that YS-121 is also a potent dual inhibitor of microsomal prostaglandin E_2_ synthase-1 (mPGES-1, IC_50_ = 3.9 µM in a cell-free assay) and 5-lipoxygenase (5-LO, IC_50_ = 4.1 µM and 6.5 µM in a cell-based and cell-free assay, respectively) with a lack of interference with COX-1 and COX-2 [7,8].

Based on those findings, more detailed investigations concerning the mechanism of mPGES-1 inhibition, the selectivity profile and the *in vivo* activity of YS-121 have been conducted [9]. The results of those experimental studies demonstrated that YS-121 inhibits mPGES-1 in a selective, concentration-dependent, reversible and noncompetitive manner. Moreover, the above-mentioned lack of interference with both isoforms of cyclooxygenase still remains, even in assays performed in human whole blood. Additionally, an anti-inflammatory efficacy of YS-121 was shown *in vivo* [9].

In summary, YS-121 is a promising drug candidate, because of its interference with many targets involved in inflammatory diseases, but there is still a huge lack of *in vivo* data concerning dosing, pharmacokinetics and effectiveness in humans.

Ongoing preclinical studies will thus necessitate huge amounts of YS-121. In medicinal chemistry the amount of compound needed for biochemical assays to get first *in vitro* data during structural optimization steps is very low. For most assays 3–4 mg are sufficient and the syntheses are planned along that required low amount of compound. But the demand for compound rises up to gram scale by conducting animal experiments to get first *in vivo* data of an *in vitro* hit. To cope with those requirements, we have optimized the synthesis of YS-121 resolving the problem of byproduct formation during that synthesis conducted in a larger scale. Furthermore, we isolated and characterized some byproducts enabling us to develop a proposal for the mechanism of that byproduct formation.

## 2. Results and Discussion

**Scheme 1:**
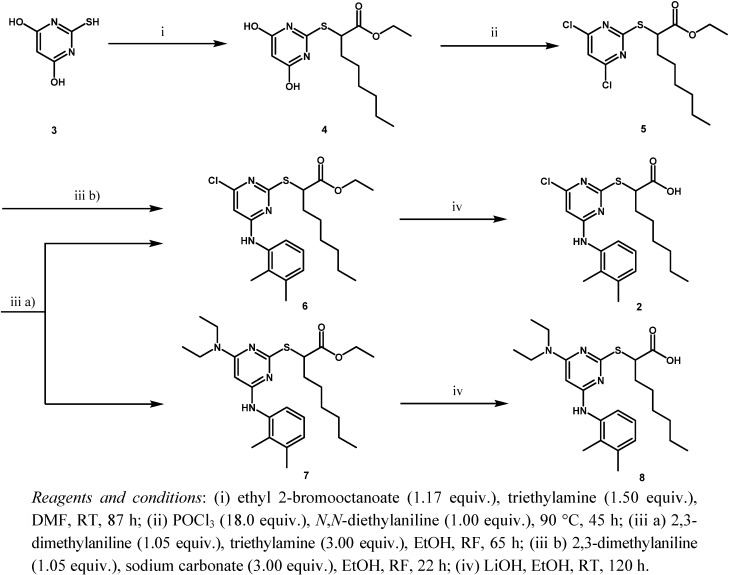
Synthesis of YS-121 **2** including byproduct formation and optimization step.

YS-121 was synthesized in a four-step reaction originally published by d’Atri *et al.* [10] and already modified by Koeberle *et al.* [8]. Here, we modified and optimized that synthesis again to make it convenient to synthesize YS-121 in amounts of a few grams. The specific conditions utilized, the problem of byproduct formation in step (iii a) and the optimization step to circumvent that byproduct formation (iii b) are illustrated in Scheme 1.

During the first step, 2-mercaptopyrimidine-4,6-diol (**3**) reacted by a nucleophilic substitution with ethyl 2-bromooctanoate in DMF in the presence of triethylamine to form the thioether derivative ethyl 2-(4,6-dihydroxypyrimidin-2-ylthio)octanoate (**4**). Chlorination with POCl_3_ and *N*,*N*-diethylaniline gave the chlorinated pyrimidine derivative ethyl 2-(4,6-dichloropyrimidin-2-ylthio)octanoate (**5**). Through a nucleophilic aromatic substitution at the pyrimidine core of **5**, one chloro group was substituted by 2,3-dimethylaniline with triethylamine in EtOH to form the monoaminated derivative ethyl 2-(4-chloro-6-(2,3-dimethylphenylamino)pyrimidin-2-ylthio)octanoate (**6**, iii a). Additionally, the disubstituted byproduct ethyl 2-(4-(diethylamino)-6-(2,3-dimethylphenylamino)pyrimidin-2-ylthio)-octanoate (**7**) was formed during that step. Changing the assisting base to sodium carbonate in step (iii b), lead to a circumvention of that byproduct formation. The last step to get to the desired compound YS-121 (**2**) consisted of a saponification of the ethyl ester derivative **6** with LiOH in EtOH to give the carboxylic acid derivative 2-(4-chloro-6-(2,3-dimethylphenylamino)-pyrimidin-2-ylthio)octanoic acid (**2**). Because **6** and **7** could not be separated during the purification procedure of step (iii a), **7** was also hydrolyzed during step (iv) to give the byproduct (2-(3-(diethylamino)-5-(2,3-dimethylphenylamino)-phenylthio)octanoic acid (**8**), which could be separated from **2** and its structure elucidated afterwards.

In comparison to Koeberle *et al.* [8], several changes have been made to optimize the reaction in general. In step (i) the relative amounts of ethyl 2-bromooctanoate and triethylamine as well as the reaction temperature were reduced in order to minimize byproduct formation initially caused by a dehydrohalogenation of ethyl 2-bromooctanoate leading to a Michael acceptor system that is able to react with nucleophiles as well. The dehydrohalogenation is a β-elimination. With regard to the desired nucleophilic substitution by **3** on ethyl 2-bromooctanoate, high temperatures and bulky bases raise the probability of β-elimination [16]. Because of the lower reaction temperature, longer reaction times were required until TLC-control showed total conversion of **3**. In step (ii) we reduced the reaction temperature to guard the product from decomposition reactions. Because of the lower reaction temperature, longer reaction times were required again until TLC-control showed total conversion of **4**. In steps (iii) and (iv) only the reaction times were expanded.

Perhaps it would have been possible to reduce the reaction times of all steps, but our aim was first and foremost to get the highest yield possible during each respective step. As described above, during structural optimization steps in medicinal chemistry, syntheses are planned to get the test compound on mg scale, which is sufficient for first *in vitro* data, but we conducted the reaction steps on a larger scale to cope with the rising demand for test compound on gram scale for further *in vivo* testing. Those higher absolute amounts of compounds during synthesis should also have expanded the reaction times.

The challenge of optimizing the synthesis of YS-121 consisted of the low yields of the different steps that were primarily based on the high level of byproduct formation. That necessitated huge reaction batches, followed by extensive purification procedures and finally led in a large part to those low yields. One of those byproducts was formed in high amounts in step (iii a). It was not described in former publications concerning that type of reaction [6,8,10,11,12,13,14], also with different aromatic amines as nucleophiles, and this forced us to modify the reaction procedure as well as the way of purification. In step (iii a) the chlorinated pyrimidine derivative **5** should be monoaminated to **6** by a nucleophilic aromatic substitution with 2,3-dimethylaniline. The reaction was conducted with triethylamine in EtOH, and the amount of the nucleophile 2,3-dimethylaniline was 1.05 equiv. with regard to **5**. After the reaction was finished, TLC-control showed the formation of at least four byproducts next to **6**. Two purification steps by column chromatography with silica gel only led to a rough purification in which three of the four byproducts could be separated from the desired product **6** in that step.

Further conversion of the mixture of step (iii a) in step (iv) again led to the isolation of two different compounds. In comparison with the ESI data of step (iii a), mass spectrometry indicated that both compounds were converted into carboxylic acids. One of them was the desired final compound **2**. We tried to separate them by column chromatography, but we were not successful. Afterwards, we switched to RP-18 silica gel for column chromatography, which finally allowed a satisfactory separation. Furthermore, after termination of the complete synthesis, we found out that purification by RP-18 silica gel for column chromatography would have been convenient for the purification in step (iii a) as well.

**Figure 3:**
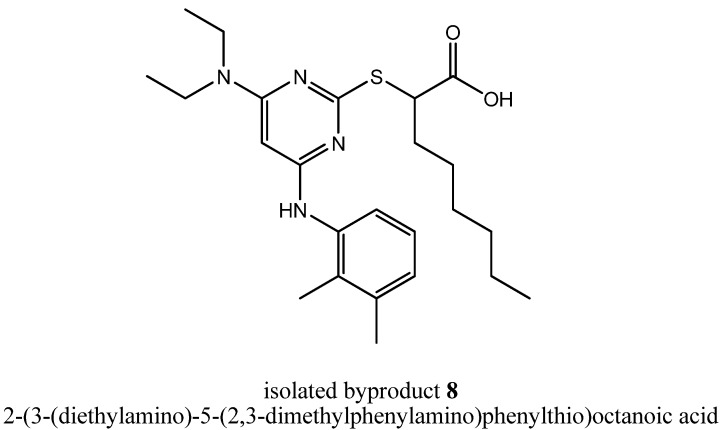
Chemical structure of the isolated byproduct **8**.

During the purification procedure of step (iv), it was possible to isolate the byproduct. The amount of the byproduct was 1:1, with regard to the amount of **2**. Characterization by the whole lineup of structure determination methods (including ^1^H-NMR, ^13^C-NMR/DEPT, COSY, ROESY, HSQC, HMBC and HRMS experiments) suggested a formation of the disubstituted compound 2-(3-(diethylamino)-5-(2,3-dimethylphenylamino)phenylthio)octanoic acid (**8**, Figure 3). Analytical data of **8** are given in the Experimental section.

As far as the formation of byproduct **8** is concerned, a disubstitution would have only been possible in step (iii a), if diethylamine or diethylamine-related reactive intermediates had been present next to 2,3-dimethylaniline as nucleophiles as well. Either that byproduct formation is the result of a diethylamine impurity of the used commercially available reagents triethylamine, 2,3-dimethylaniline or EtOH in step (iii a) or it is a further indication that triethylamine could react as a nucleophile during nucleophilic aromatic substitution reactions at heteroaromatic halides as previously described by Matsumoto *et al.* [15].

**Figure 4:**
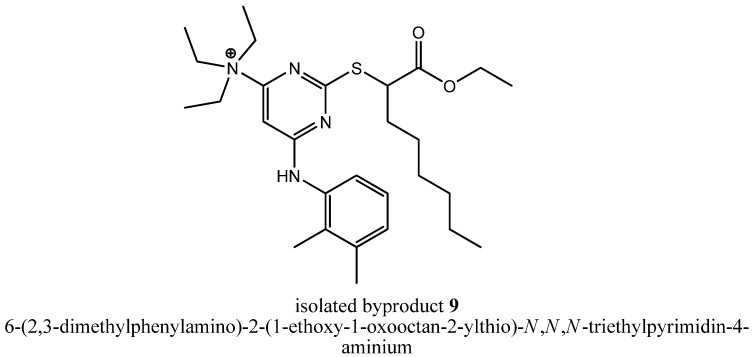
Chemical structure of the isolated byproduct **9**.

To get deeper insights into the mechanism leading to the formation of byproduct **8**, we repeated step (iii a) with triethylamine as assisting base and could reproduce that byproduct formation in two reaction batches reacted independently from each other. Afterwards, we tried to isolate other byproducts of that reaction that could tell us something about the mechanism leading to **8**. In fact, we were successful, because we could isolate the stable intermediate 6-(2,3-dimethylphenylamino)-2-(1-ethoxy-1-oxooctan-2-ylthio)-*N*,*N*,*N*-triethylpyrimidin-4-aminium (**9**, Figure 4), the precursor of compound **7**.

**Figure 5:**
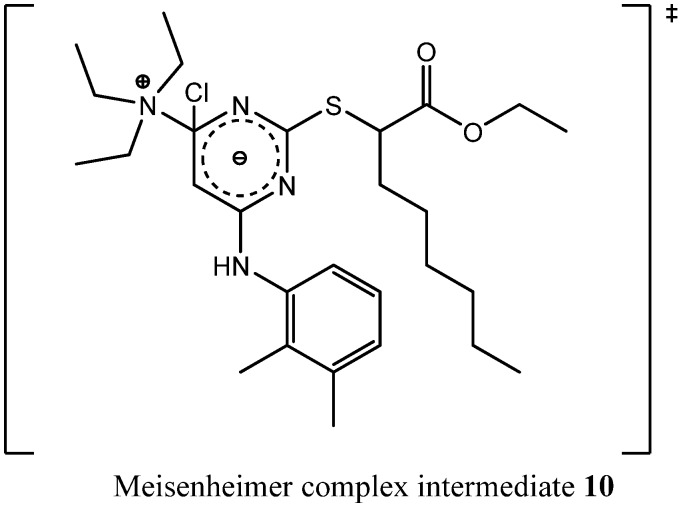
Chemical structure of the Meisenheimer complex intermediate **10**.

Compound **9** is a quaternary ammonium cation. Furthermore, both chlorines were already substituted, so the formation of **9** probably lead in the first stage to a Meisenheimer complex intermediate **10** (Figure 5) that was formed by a nucleophilic attack of triethylamine leading in second stage to an elimination of a chloride ion.

**Figure 6:**
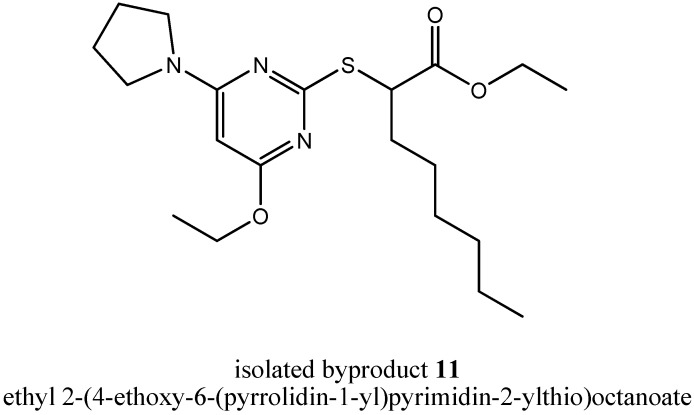
Chemical structure of the isolated byproduct **11**.

Additionally, other byproducts were formed in step (iii a), but we were not able to separate them satisfactorily. Because the insights into the mechanism of byproduct formation were still not sufficient, we repeated step (iii a) again, now with *N*-methylpyrrolidine as assisting base as a member of cyclic tertiary alkylamines. Again, we tried to isolate byproducts giving us new insights into the mechanism of byproduct formation and again, we were successful. We could isolate two byproducts telling us a lot about the mechanism. First, we could isolate the byproduct ethyl 2-(4-ethoxy-6-(pyrrolidin-1-yl)pyrimidin-2-ylthio)octanoate (**11**, Figure 6).

In comparison to compound **5**, several changes took place at the pyrimidine core. Both chlorines were substituted, the first by *N*-methylpyrrolidine and the second by the solvent ethanol. During byproduct formation, *N*-methylpyrrolidine lost its methyl group. But that byproduct as well as byproduct **8** could not tell us how in both cases the quaternary ammonium cation had lost its fourth carbon residue leading to a *N,N*-dialkylaminyl substituent at the pyrimidine core. In summary, during formation of byproduct **11**, one equivalent of methyl chloride and one equivalent of hydrochloric acid were eliminated.

**Figure 7:**
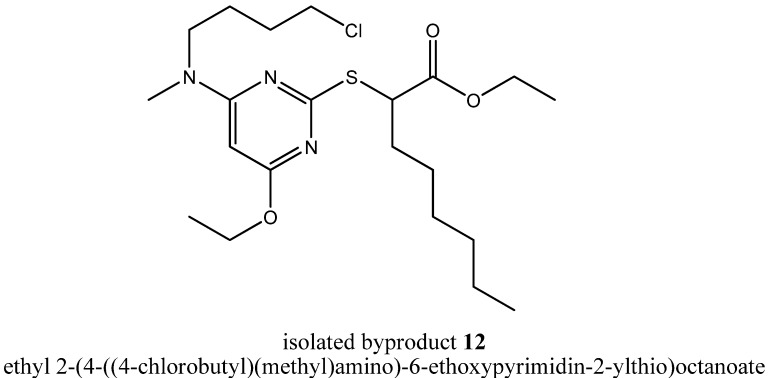
Chemical structure of the isolated byproduct **12**.

A deeper insight into the incidents taking place at the pyrrolidinyl residue was provided by the isolated byproduct ethyl 2-(4-((4-chlorobutyl)(methyl)amino)-6-ethoxypyrimidin-2-ylthio)octanoate (**12**, Figure 7).

**Figure 8:**
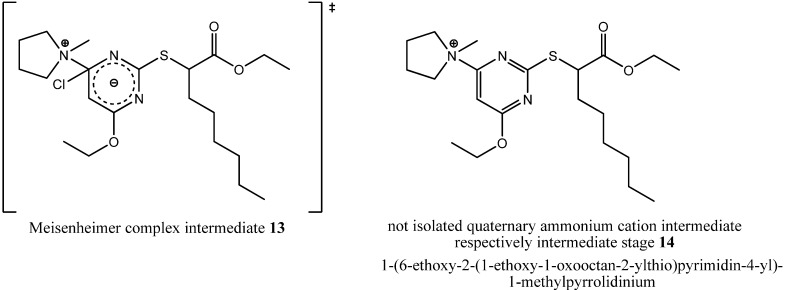
Chemical structures of not isolated hypothetical intermediates of the respective intermediate stages during reaction with *N*-methylpyrrolidine.

During formation of byproduct **12** the pyrrolidine ring was opened but no carbon residue was eliminated. In summary, only one equivalent of hydrochloric acid was eliminated through a solvolytic reaction with ethanol, but the terminally chlorinated *n*-butyl residue as well as the methyl residue of the *N*,*N*-dialkylaminyl substituent at the pyrimidine core provided evidence for the ring opening mechanism. That ring opening probably had happened either through a nucleophilic attack of a chloride ion during the state of a Meisenheimer complex intermediate **13** as a concerted process together with the elimination of the chloride ion from the pyrimidine core or during the state of a quaternary ammonium cation such as 1-(6-ethoxy-2-(1-ethoxy-1-oxooctan-2-ylthio)pyrimidin-4-yl)-1-methylpyrrolidinium (**14**, Figure 8) as a non-concerted process.

We could not isolate either of the two intermediates, but because we could isolate byproduct **9**, so the non-concerted process of a nucleophilic attack of a chloride ion after elimination from the pyrimidine core seems to be more likely. The solvolytic reaction at the pyrimidine core also stresses that last presumption. Only during the stage of the quaternary ammonium cation, a nucleophilic attack of the solvent is possible and catalyzed, because *N*-methylpyrrolidine is a good leaving group in that case. The potential of tertiary amines to catalyze nucleophilic substitution reactions at aromatic heterocycles was already described in the literature [17]. The fact that we could not isolate solvolytic reaction byproducts from the reaction with triethylamine could be due to the steric hindrance at the tertiary ammonium cation **9** preventing a nucleophilic attack of the solvent.

Another interesting fact is that during reaction with *N*-methylpyrrolidine we could not isolate any amount of desired compound **6** at all, although we had not changed the relative amounts of educts. That suggests a higher nucleophilic potential of *N*-methylpyrrolidine as well as a higher potential of *N*-methylpyrrolidine to catalyze solvolytic reactions at the chlorinated pyrimidine core in comparison to triethylamine.

**Scheme 2:**
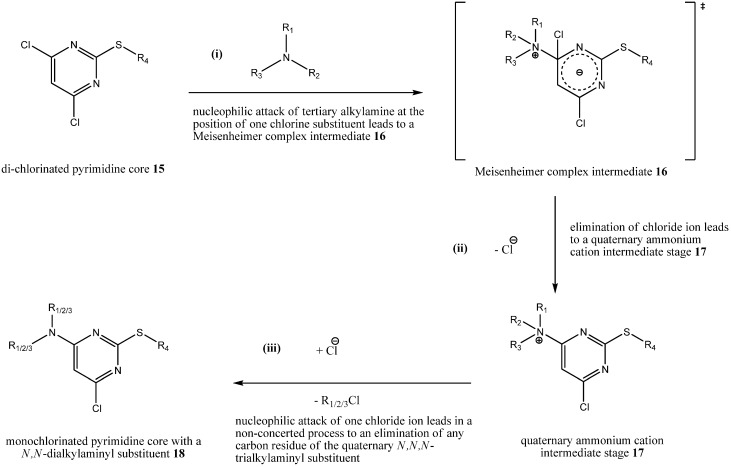
Postulated mechanism of byproduct formation.

To sum it up, the following Scheme 2 depicts our proposal for the mechanism of byproduct formation based on the isolated and characterized byproducts **8**, **9**, **11** and **12** during step (iii a) with two different tertiary alkylamines as assisting bases:

(i) A nucleophilic attack of a tertiary alkylamine at a 4,6-di-chlorinated pyrimidine core **15** leads to a Meisenheimer complex intermediate **16**; (ii) afterwards, a chloride ion is eliminated leading through rearomatization to a quaternary ammonium cation intermediate stage **17**; (iii) finally, a nucleophilic attack by a chloride ion at any carbon residues of the quaternary *N*,*N*,*N*-trialkylaminyl substituent of **17** leads to a monochlorinated pyrimidine core with a *N*,*N*-dialkylaminyl substituent **18**.

With the isolation of byproduct **9** and the support from isolated byproducts **11** and **12** we can state that formation of byproduct **8** during step (iii a) was due to a direct nucleophilic attack of the tertiary alkylamine triethylamine at the chlorinated pyrimidine core of **5**. Because triethylamine and other tertiary alkylamines are broadly used as assisting bases during nucleophilic aromatic substitution reactions due to their low nucleophilicity, our results put their use into question during that type of reactions.

As stated above, the formation of that sort of byproducts was not described in former publications about that type of reaction showing the synthesis of pirinixic acid derivatives. One cause of the fact that byproduct **8** is apparently formed exclusively during reaction batches on a larger scale could probably be the higher concentration of triethylamine during step (iii a) that has raised the probability of a nucleophilic attack of triethylamine at the chlorinated pyrimidine core. Therefore, we think that it could be useful to lower the concentration of triethylamine in step (iii a) or to change the assisting base triethylamine to sodium carbonate as depicted in step (iii b) and as previously described by Rau *et al.* [6] and Popescu *et al.* [11] to avoid the formation of byproduct **8**.

We checked that last proposal for effectiveness during step (iii b) and indeed, byproducts were formed to a lesser extent. That made the purification procedure less complex. Therefore, we succeeded in optimizing reaction step (iii a) by changing the assisting base triethylamine to sodium carbonate as depicted in step (iii b). So, all forms of reactions utilizing inorganic assisting bases during nucleophilic aromatic substitution reactions at heteroaromatic halides seem to be more suitable than reactions using tertiary alkylamines as assisting bases.

## 3. Experimental

### 3.1. General

*Compounds and Chemistry*. All commercial chemicals and solvents were of reagent grade and were used without further purification. For step (i), commercially available triethylamine was distilled after refluxing over KOH. All reactions were carried out in an argon atmosphere and compounds **2**, **4**, **5**, **6**, **8**, **9**, **11** and **12** were permanently stored under argon after their purification. The structures of compounds **2**, **4**, **5**, **6**, **8**, **9**, **11** and **12** were confirmed by ^1^H-NMR spectroscopy and mass spectrometry (ESI). The structures of final compound **2** and byproducts, **8**, **9**, **11**, and **12** were additionally confirmed by ^13^C-NMR spectroscopy and in part by high-resolution mass spectrometry (HRMS). Furthermore, the purity of compound **2** was determined by combustion analysis and high pressure liquid chromatography (HPLC) analysis and was 99%.

^1^H- and ^13^C-NMR spectra were measured in DMSO-*d*_6_ on a Bruker AM 250, DPX 250, AV 300 or AV 400 spectrometer (Bruker, Rheinstetten, Germany). For correct correlation of ^13^C-NMR signals to the respective carbon atom in compound **2** and for structure determination of byproduct 7 in general, ^13^C distortionless enhancement by polarization transfer (DEPT), ^1^H-^1^H correlation spectroscopy (COSY), ^1^H-^1^H rotating frame nuclear overhauser effect spectroscopy (ROESY), ^1^H-^13^C heteronuclear single quantum coherence (HSQC) and ^1^H-^13^C heteronuclear multiple bond correlation (HMBC) experiments were conducted. They were also performed on the spectrometers mentioned above. Chemical shifts are reported in parts per million (ppm) using tetramethylsilane (TMS) as an internal standard. The following abbreviations were used to explain the multiplicities: s = singlet, d = doublet, t = triplet, q = quartet, m = multiplet, br = broad. Mass spectra were obtained on a Fisons Instruments VG Platform II spectrometer measuring in the positive- or negative-ion mode (ESI-MS system, Fisons Instruments, Manchester, Great Britain). High-resolution mass spectra (HRMS) were recorded on a Thermo Scientific MALDI LTQ Orbitrap XL (Thermo Fisher Scientific, Waltham, MA, USA). Combustion analysis was performed by the Microanalytical Laboratory of the Institute of Organic Chemistry and Chemical Biology, Goethe University Frankfurt, on a Foss Heraeus CHN-O-rapid elemental analyzer (Heraeus, Hanau, Germany). High pressure liquid chromatography (HPLC) analysis was carried out on a Waters instrument (Waters, Milford, CT, USA) consisting of a Waters 600 controller, an In-Line Degasser AF, a Waters 717plus autosampler and a Waters 2487 dual λ absorbance detector. The utilized detection wavelengths were 247, 254 and 290 nm. The column used was a MultoHigh Phenyl-5 µm column, 250 × 4 mm (CS Chromatographie, Langerwehe, Germany). The mobile phase gradient used consisted of 2 phases (MeOH = phase A, Milli-Q distillate 0.1% formic acid = phase B) starting with 10% phase A up to 100% phase A within 45 min. The used flow rate was 1 mL/min and the separation temperature was set at 25 °C. Reactions were monitored by thin-layer chromatography that was carried out on Merck TLC silica gel plates 60 F_254_ and RP-18 F_254 s_ (Merck, Darmstadt, Germany) using UV light as visualizing agent. Macherey Nagel silica gel 60, particle size 0.040–0.063 mm/230–400 mesh ASTM (Macherey Nagel, Düren, Germany) and Sigma-Aldrich octadecyl-functionalized silica gel, particle size 200–400 mesh, extent of loading: 20–22% (Sigma-Aldrich, St. Louis, MO, USA) were used for column chromatography. The synthesis of YS-121 followed the route originally published by d’Atri *et al.* [10] and already modified by Koeberle *et al.* [8]. That synthesis was modified and optimized again in some cases to be convenient to resolve the problem of byproduct formation during that synthesis conducted in a larger scale.

### 3.2. Ethyl 2-(4,6-Dihydroxypyrimidin-2-ylthio)octanoate *(**4**)*

*Step (i).* 2-Mercaptopyrimidine-4,6-diol (**3**; 30.0 g, 208 mmol, 1.00 equiv.) was dissolved in anhydrous DMF (150 mL) under heating. Triethylamine (31.5 g, 312 mmol, 1.50 equiv.) was added at 23 °C through a dripping funnel, followed by ethyl 2-bromooctanoate (61.4 g, 244 mmol, 1.17 equiv.). After stirring at room temperature for 87 h (TLC control), the DMF was evaporated under reduced pressure. The residue was suspended in water and extracted with EtOAc. The combined organic layers were washed with brine, dried with MgSO_4_, filtered and evaporated under reduced pressure. The crude product was recrystallized from EtOAc/*n*-hexane to give **4** (27.3 g, 42%) as a white solid. R*_f_* = 0.64 (silica gel, EtOAc-MeOH 3:1); ^1^H-NMR [250.13 MHz, (CD_3_)_2_SO]: δ = 0.86 (t, 3H, C*H*_3_-Hex, *J* = 6.8 Hz), 1.17 (t, 3H, OCH_2_C*H*_3_, *J* = 7.0 Hz), 1.25–1.44 (m, 8H, C*H*_2_-Hex), 1.73–1.94 (m, 2H, C*H*_2_-Hex), 4.14 (q, 2H, OC*H*_2_, *J* = 7.3 Hz), 4.52 (t, 1H, SC*H*, *J* = 7.3 Hz), 5.25 (s, 1H, Pyr-5*H*), 11.81 (br s, 2H, O*H*); MS (ESI+): *m/e* = 315.1 [M + H]^+^.

### 3.3. Ethyl 2-(4,6-Dichloropyrimidin-2-ylthio)octanoate *(**5**)*

*Step (ii).* POCl_3_ (219 g, 1.43 mol, 18.0 equiv.) was added through a dripping funnel at 0 °C to the thioether derivative **4** (25.0 g, 79.5 mmol, 1.00 equiv.) resulting from step (i), followed by *N*,*N*-diethylaniline (11.9 g, 79.5 mmol, 1.00 equiv.) at 0 °C. That mixture was stirred at 90 °C for 45 h (TLC control). Then, excess POCl_3_ was distilled off at atmospheric pressure, and the resulting oil was poured onto crushed ice. The aqueous solution was extracted with EtOAc, and the combined organic layers were washed with diluted HCl, saturated NaHCO_3_ solution and brine. Then, the organic layer was dried over MgSO_4_, filtered and evaporated under reduced pressure. The crude product was purified by column chromatography (silica gel, *n*-hexane-EtOAc 5:1) to give **5** (24.5 g, 88%) as a red oil. R*_f_* = 0.6 (silica gel, n-hexane-EtOAc 5:1); ^1^H-NMR [400.13 MHz, (CD_3_)_2_SO]: δ = 0.86 (t, 3H, C*H*_3_-Hex, *J* = 7.2 Hz), 1.20 (t, 3H, OCH_2_C*H*_3_, *J* = 6.8 Hz), 1.24–1.34 (m, 6H, C*H*_2_-Hex), 1.36–1.43 (m, 2H, C*H*_2_-Hex), 1.79–1.97 (m, 2H, C*H*_2_-Hex), 4.15 (q, 2H, OC*H*_2_, *J* = 7.2 Hz), 4.37 (t, 1H, SC*H*, *J* = 7.2 Hz), 7.75 (s, 1H, Pyr-5*H*); MS (ESI+): *m/e* = 351.1 [M + H]^+^.

### 3.4. Ethyl 2-(4-Chloro-6-(2,3-dimethylphenylamino)pyrimidin-2-ylthio)octanoate *(**6**)* and 2-(4-Chloro-6-(2,3-dimethylphenylamino)pyrimidin-2-ylthio)octanoic Acid *(**2**)*

*Step (iii a).* A solution of the chlorinated pyrimidine derivative **5** (18.7 g, 53.2 mmol, 1.00 equiv.) obtained from step (ii), triethylamine (16.2 g, 160 mmol, 3.00 equiv.) and 2,3-dimethylaniline (6.78 g, 55.9 mmol, 1.05 equiv.) in EtOH (62.5 mL) was refluxed under stirring for 65 h (TLC control). Then, EtOH and excess triethylamine were evaporated under reduced pressure. The residue was dissolved in EtOAc, and the organic layer was extracted with diluted HCl, saturated NaHCO_3_ solution and brine. Then, the organic layer was dried over MgSO_4_, filtered and evaporated under reduced pressure. The crude product was roughly purified by column chromatography (silica gel, *n*-hexane-EtOAc 10:1) to give a mixture of the desired compound **6** together with one byproduct as a yellow oil (14.0 g). MS (ESI+): *m/e* = 436.2 [M + H]^+^, byproduct: MS (ESI+): *m/e* = 473.3 [M + H]^+^. That mixture was used for step (iv) without further purification.

After completion of step (iv), further purification of 500 mg of that mixture by column chromatography (RP-18 silica gel, acetonitrile-water 4:1) gave the desired compound **6** (210 mg) as a yellow oil. Therefore, purification by RP-18 silica gel is already useful and applicable at that stage of synthesis. By extrapolating the result of RP-18 column chromatography of 500 mg up to 14.0 g, a yield of 5.88 g, 25% of **6** could have been achievable. R*_f_* = 0.2 (silica gel, *n*-hexane-EtOAc 10:1); ^1^H-NMR (250.13 MHz, (CD_3_)_2_SO): δ = 0.83 (t, 3H, C*H*_3_-Hex, *J* = 7.0 Hz), 1.15 (t, 3H, OCH_2_C*H*_3_, *J* = 7.0 Hz), 1.16–1.34 (m, 8H, C*H*_2_-Hex), 1.63–1.84 (m, 2H, C*H*_2_-Hex), 2.05 (s, 3H, Ph-2-C*H*_3_), 2.26 (s, 3H, Ph-3-C*H*_3_), 4.08 (q, 2H, OC*H*_2_, *J* = 7.3 Hz), 4.25 (t, 1H, SC*H*, *J* = 7.3 Hz), 6.20 (s, 1H, Pyr-5*H*), 7.06-7.13 (m, 3H, Ph-4,5,6-*H*), 9.51 (br s, 1H, N*H*); MS (ESI+): *m/e* = 436.2 [M + H]^+^.

*Step (iv).* The mixture of **6** and one byproduct (13.5 g) obtained in step (iii a) was dissolved in EtOH (420 mL) at room temperature. An approximate excess of LiOH (2.61 g, 109 mmol) was added, and the resulting mixture was stirred at room temperature for 120 h (TLC control). Then, EtOH was removed under reduced pressure, and the obtained residue was dissolved in water under heating, while low amounts of MeOH were added. The solution was acidified with diluted HCl, and the precipitate was filtered, washed to neutrality with water and then with n-hexane. A solution of the precipitate in EtOAc was dried over MgSO_4_, filtered and evaporated under reduced pressure. The crude product was purified by column chromatography (RP-18 silica gel, acetonitrile-water 4:1). Finally a solution of the purified product in EtOAc was extracted with diluted HCl, washed to neutrality with water and was dried over MgSO_4_. After filtration, the solvent was evaporated under reduced pressure and the product was precipitated from EtOAc/*n*-hexane. After two weeks at 26 °C, the solid product was filtered and was washed with n-hexane. In the end 2.00 g of the carboxylic acid derivative **2** were obtained as a pale yellow solid. By including the extrapolated content of **6** in the mixture used in that step, a yield of 38% was achieved. R*_f_* = 0.4 (silica gel, EtOAc); ^1^H-NMR [250.13 MHz, (CD_3_)_2_SO]: δ = 0.85 (t, 3H, C*H*_3_-Hex, *J* = 6.3 Hz), 1.10–1.37 (m, 8H, C*H*_2_-Hex), 1.59–1.91 (m, 2H, C*H*_2_-Hex), 2.08 (s, 3H, Ph-2-C*H*_3_), 2.28 (s, 3H, Ph-3-C*H*_3_), 4.23 (br t, 1H, SC*H*), 6.21 (s, 1H, Pyr-5*H*), 7.08–7.16 (m, 3H, Ph-4,5,6-*H*), 9.51 (br s, 1H, N*H*), 12.82 (br s, 1H, COO*H*); ^13^C-NMR [62.90 MHz, (CD_3_)_2_SO]: δ = 13.85 (*C*H_3_-Hex), 13.99 (Ph-2-*C*H_3_), 20.06 (Ph-3-*C*H_3_), 21.90 (*C*H_2_-Hex), 26.38 (*C*H_2_-Hex), 28.08 (*C*H_2_-Hex), 30.94 (*C*H_2_-Hex), 31.95 (*C*H_2_-Hex), 46.93 (S*C*H), 98.90 (Pyr-5-*C*), 123.99 (Ph-6-*C*), 125.67 (Ph-5-*C*), 127.83 (Ph-4-*C*), 132.33 (Ph-2-*C*), 135.61 (Ph-3-*C*), 137.59 (Ph-1-*C*), 157.73 (Pyr-4-*C*), 162.16 (Pyr-2-*C*), 169.63 (Pyr-6-*C*), 172.47 (*C*OOH); MS (ESI+): *m/e* = 408.1 [M + H]^+^; HRMS (MALDI): calcd. for C_20_H_27_ClN_3_O_2_S^+^ [M + H]^+^ = 408.1507, found 408.1509; Anal. Calcd. C_20_H_26_ClN_3_O_2_S C, H, N: Calc. C 58.88, H 6.42, N 10.30, found C 59.12, H 6.52, N 10.15.

### 3.5. Analytical data of (2-(3-(Diethylamino)-5-(2,3-dimethylphenylamino)phenylthio)octanoic Acid *(**8**)*

During purification procedure of step (iv), **8** could be isolated as well as a pale, yellow solid. R*_f_* = 0.6 (silica gel, EtOAc); ^1^H-NMR [250.13 MHz, (CD_3_)_2_SO]: δ = 0.85 (t, 3H, C*H*_3_-Hex, *J* = 6.8 Hz), 1.05 (t, 6H, N(CH_2_C*H*_3_)_2_, *J* = 7.0 Hz), 1.19–1.41 (m, 8H, C*H*_2_-Hex), 1.69–1.88 (m, 2H, C*H*_2_-Hex), 2.08 (s, 3H, Ph-2-C*H*_3_), 2.26 (s, 3H, Ph-3-C*H*_3_), 3.27–3.43 (m, 4H, N(C*H*_2_CH_3_)_2_), 4.25 (t, 1H, SC*H*, *J* = 7.5), 5.19 (s, 1H, Pyr-5*H*), 6.98–7.15 (m, 3H, Ph-4,5,6-*H*), 8.38 (br s, 1H, N*H*), 12.74 (br s, 1H, COO*H*); ^13^C- NMR [62.90 MHz, (CD_3_)_2_SO]: δ = 12.79 (N(CH_2_*C*H_3_)_2_), 13.84 (*C*H_3_-Hex), 14.01 (Ph-2-*C*H_3_), 20.16 (Ph-3-*C*H_3_), 21.92 (*C*H_2_-Hex), 26.80 (*C*H_2_-Hex), 28.30 (*C*H_2_-Hex), 31.07 (*C*H_2_-Hex), 32.42 (*C*H_2_-Hex), 41.49 (N(*C*H_2_CH_3_)_2_), 46.96 (S*C*H), 77.39 (Pyr-5-*C*), 123.43 (Ph-6-*C*), 125.32 (Ph-5-*C*), 126.38 (Ph-4-*C*), 131.59 (Ph-2-*C*), 137.13 (Ph-3-*C*), 137.52 (Ph-1-*C*), 160.42 (Pyr-4-*C*), 161.80 (Pyr-2-*C*), 167.63 (Pyr-6-*C*), 173.29 (*C*OOH); MS (ESI+): *m/e* = 445.3 [M + H]^+^; HRMS (MALDI): calcd. for C_24_H_37_N_4_O_2_S^+^ [M + H]^+^ = 445.2632, found 445.2635.

### 3.6. 6-(2,3-Dimethylphenylamino)-2-(1-ethoxy-1-oxooctan-2-ylthio)-N,N,N-triethylpyrimidin-4-aminium *(**9**)*

A solution of the chlorinated pyrimidine derivative **5** (400 mg, 1.14 mmol, 1.00 equiv.) obtained from step (ii), triethylamine (346 mg, 3.42 mmol, 3.00 equiv.) and 2,3-dimethylaniline (145 mg, 1.20 mmol, 1.05 equiv.) in EtOH (1.33 mL) was refluxed under stirring for 14 h (TLC control). Then, EtOH and excessive triethylamine were evaporated under reduced pressure. The residue was dissolved in EtOAc, and the organic layer was extracted with diluted HCl, saturated NaHCO_3_ solution and brine. Then, the organic layer was dried over MgSO_4_, filtered and evaporated under reduced pressure. Compound **9** was precipitated from EtOAc/*n*-hexane at 26 °C to give **9** (70.0 mg, 12%) as a colorless solid. ^1^H-NMR [400.13 MHz, (CD_3_)_2_SO]: δ = 0.86 (t, 3H, C*H*_3_-Hex, *J* = 6.7 Hz), 1.11–1.39 (m, 20H, N(CH_2_C*H*_3_)_3_, OCH_2_C*H*_3_, C*H*_2_-Hex), 1.71–1.89 (m, 2H, C*H*_2_-Hex), 2.10 (s, 3H, Ph-2-C*H*_3_), 2.30 (s, 3H, Ph-3-C*H*_3_), 3.65–3.82 (m, 6H, N(C*H*_2_CH_3_)_3_), 4.04–4.16 (m, 2H, OC*H*_2_), 4.30 (br t, 1H, SC*H*), 6.91–7.24 (m, 4H, Pyr-5*H*, Ph-4,5,6-*H*), 10.22 (s, 1H, N*H*); ^13^C-NMR [75.44 MHz, (CD_3_)_2_SO]: δ = 7.68 (N(CH_2_*C*H_3_)_3_), 13.82 (*C*H_3_-Hex), 13.92 (OCH_2_*C*H_3_), 14.18 (Ph-2-*C*H_3_), 20.08 (Ph-3-*C*H_3_), 21.85 (*C*H_2_-Hex), 26.39 (*C*H_2_-Hex), 28.08 (*C*H_2_-Hex), 30.87 (*C*H_2_-Hex), 30.95 (*C*H_2_-Hex), 47.07 (S*C*H), 53.41 (N(*C*H_2_CH_3_)_3_), 61.05 (COO*C*H_2_), 95.39 (Pyr-5-*C*), 123.57 (Ph-6-*C*), 125.63 (Ph-5-*C*), 127.94 (Ph-4-*C*), 131.92 (Ph-2-*C*), 135.42 (Ph-3-*C*), 137.59 (Ph-1-*C*), 159.10 (Pyr-4-*C*), 162.76 (Pyr-2-*C*), 170.26 (Pyr-6-*C*), 171.21 (*C*OOCH_2_CH_3_); MS (ESI+): *m/e* = 502.3.

### 3.7. Ethyl 2-(4-Ethoxy-6-(pyrrolidin-1-yl)pyrimidin-2-ylthio)octanoate *(**11**)* and Ethyl 2-(4-((4-chloro-butyl)(methyl)amino)-6-ethoxypyrimidin-2-ylthio)octanoate *(**12**)*

A solution of the chlorinated pyrimidine derivative **5** (200 mg, 0.569 mmol, 1.00 equiv.) obtained from step (ii), *N*-methylpyrrolidine (145 mg, 1.71 mmol, 3.00 equiv.) and 2,3-dimethylaniline (72.4 mg, 0.598 mmol, 1.05 equiv.) in EtOH (0.667 mL) was refluxed under stirring for 21 h (TLC control). Then, EtOH and excessive *N*-methylpyrrolidine were evaporated under reduced pressure. The residue was dissolved in EtOAc, and the organic layer was extracted with diluted HCl, saturated NaHCO_3_ solution and brine. Then, the organic layer was dried over MgSO_4_, filtered and evaporated under reduced pressure. The crude product was roughly purified by column chromatography (silica gel, *n*-hexane-EtOAc 20:1) to give a mixture of compound **11** and compound **12**. To separate both compounds a second column chromatography step followed using silica gel and an eluent gradient of *n*-hexane-EtOAc from 50:1 to 20:1 to give **11** (8.0 mg, 4%) as a colorless oil and **12** (10.0 mg, 4%) as a light yellow oil.

*Compound* **11**. R*_f_* = 0.4 (silica gel, n-hexane-EtOAc 10:1); ^1^H-NMR [400.13 MHz, (CD_3_)_2_SO]: δ = 0.86 (t, 3H, C*H*_3_-Hex, *J* = 6.8 Hz), 1.17 (t, 3H, COOCH_2_C*H*_3_, *J* = 7.1 Hz), 1.25–1.43 (m, 11H, ArOCH_2_C*H*_3_, C*H*_2_-Hex), 1.75–1.99 (m, 6H, C*H*_2_-Hex, Pyrrolidine-2-C*H_2_*, Pyrrolidine-3-C*H_2_*), 3.24–3.42 (m, 4H, Pyrrolidine-1-C*H_2_*, Pyrrolidine-4-C*H_2_*), 4.06–4.16 (m, 2H, COOC*H*_2_), 4.21–4.26 (m, 2H, ArOC*H*_2_), 4.35 (t, 1H, SC*H*, *J* = 7.3 Hz), 5.42 (s, 1H, Pyr-5*H*); ^13^C-NMR [62.90 MHz, (CD_3_)_2_SO]: δ = 13.80 (*C*H_3_-Hex), 13.93 (COOCH_2_*C*H_3_), 14.43 (ArOCH_2_*C*H_3_), 21.89 (*C*H_2_-Hex), 24.62 (Pyrrolidine-2-*C*H_2_, Pyrrolidine-3-*C*H_2_), 26.62 (*C*H_2_-Hex), 28.11 (*C*H_2_-Hex), 30.93 (*C*H_2_-Hex), 31.37 (*C*H_2_-Hex), 46.07 (Pyrrolidine-1-*C*H_2_, Pyrrolidine-4-*C*H_2_), 46.88 (S*C*H), 60.70 (COO*C*H_2_),61.43 (ArO*C*H_2_), 81.23 (Pyr-5-*C*), 161.02 (Pyr-2-*C*), 167.38 (Pyr-4-*C*), 168.39 (Pyr-6-*C*), 171.72 (*C*OOCH_2_CH_3_); MS (ESI+): *m/e* =396.2.

*Compound* **12**. R*_f_* = 0.3 (silica gel, n-hexane-EtOAc 10:1); ^1^H-NMR [400.13 MHz, (CD_3_)_2_SO]: δ = 0.86 (t, 3H, C*H*_3_-Hex, *J* = 6.8 Hz), 1.17 (t, 3H, COOCH_2_C*H*_3_, *J* = 7.1 Hz), 1.25–1.43 (m, 11H, ArOCH_2_C*H*_3_, C*H*_2_-Hex), 1.59–1.99 (m, 6H, C*H*_2_-Hex, NCH_2_C*H_2_*C*H_2_*CH_2_Cl), 2.95 (s, 1H, NC*H_3_*), 3.43–3.59 (m, 2H, NC*H_2_*CH_2_CH_2_CH_2_Cl), 4.06–4.16 (m, 2H, COOC*H*_2_), 4.21–4.26 (m, 2H, ArOC*H*_2_), 4.34 (t, 1H, SC*H*, *J* = 7.3 Hz), 5.60 (s, 1H, Pyr-5*H*); ^13^C-NMR [62.90 MHz, (CD_3_)_2_SO]: δ = 13.80 (*C*H_3_-Hex), 13.93 (COOCH_2_*C*H_3_), 14.43 (ArOCH_2_*C*H_3_), 21.89 (*C*H_2_-Hex), 24.13 (NCH_2_*C*H_2_CH_2_CH_2_Cl), 26.62 (*C*H_2_-Hex), 28.09 (*C*H_2_-Hex), 29.36 (NCH_2_CH_2_*C*H_2_CH_2_Cl), 30.93 (*C*H_2_-Hex), 31.32 (*C*H_2_-Hex), 35.13 (N*C*H_3_), 45.09 (NCH_2_CH_2_CH_2_*C*H_2_Cl), 46.79 (S*C*H), 47.48 (N*C*H_2_CH_2_CH_2_CH_2_Cl), 60.74 (COO*C*H_2_), 61.51 (ArO*C*H_2_), 80.63 (Pyr-5-*C*), 162.83 (Pyr-2-*C*), 167.31 (Pyr-4-*C*), 168.98 (Pyr-6-*C*), 171.71 (*C*OOCH_2_CH_3_); MS (ESI+): *m/e* = 446.2.

### 3.8. Ethyl 2-(4-Chloro-6-(2,3-dimethylphenylamino)pyrimidin-2-ylthio)octanoate *(**6**)*

*Step (iii b).* A solution of the chlorinated pyrimidine derivative **5** (200 mg, 0.569 mmol, 1.00 equiv.) obtained from step (ii), sodium carbonate (180 mg, 1.71 mmol, 3.00 equiv.) and 2,3-dimethylaniline (72.4 mg, 0.598 mmol, 1.05 equiv.) in EtOH (1.33 mL) was refluxed under stirring for 22 h (TLC control). Then, excessive sodium carbonate was filtrated and EtOH was evaporated under reduced pressure. The residue was dissolved in EtOAc, and the organic layer was extracted with diluted HCl, saturated NaHCO_3_ solution and brine. Then, the organic layer was dried over MgSO_4_, filtered and evaporated under reduced pressure. The crude product was purified by column chromatography (silica gel, n-hexane-EtOAc 10:1) to give compound **6** (92 mg, 35%) as a yellow oil. For analytical data of **6**, see 3.4.

## 4. Conclusions

Here, we have demonstrated that syntheses conducted in a larger scale can generate problems and byproducts that did not appear at these syntheses at the original small scale, but we also show that it is possible to resolve these problems by a change in the purification procedure or reagents used as well as in the way of synthesis and thus obtained clean compounds. By conducting the described synthesis, it was possible to generate up to 2.00 g of YS-121 (**2**) as a pure compound for use in ongoing preclinical studies. By a change of the assisting base triethylamine to sodium carbonate in step (iii b), we succeeded in optimizing that step. Byproducts were formed to a lesser extent and the purification procedure was dramatically simplified.

The reaction times are probably reducible, but our aim was first and foremost to get the highest yield possible during each respective step. After having conducted several reactions with two different assisting bases in step (iii a) and after having isolated and characterized different byproducts formed during those reactions, we could develop a proposal for the mechanism of byproduct formation. Therefore, we could clearly identify triethylamine as the reason for the formation of byproduct **7** during step (iii a) that was isolated later, in step (iv), after saponification, as **8**.

Additionally, we could show that also other tertiary alkylamines could act as nucleophiles during a nucleophilic aromatic substitution reaction at a heteroaromatic halide. Such a formation of byproducts resulting from an initial nucleophilic attack of a tertiary alkylamine and a following elimination of one alkyl residue leading to a *N*,*N*-dialkylaminyl substituent at the heteroaromatic core was not described before during the used conditions. That unexpected fact can be very useful for reaction improvement of other nucleophilic aromatic substitution reactions at heteroaromatic halides in general.
